# stepRNA: Identification of Dicer cleavage signatures and passenger strand lengths in small RNA sequences

**DOI:** 10.3389/fbinf.2022.994871

**Published:** 2022-11-21

**Authors:** Ben Murcott, Rebecca J. Pawluk, Anna V. Protasio, Ruth Y. Akinmusola, Dominika Lastik, Vicky L. Hunt

**Affiliations:** ^1^ Life Sciences Department, University of Bath, Bath, United Kingdom; ^2^ Department of Pathology, University of Cambridge, Cambridge, United Kingdom

**Keywords:** small RNA, Dicer, small-interfering RNA, non-coding RNA, RNA processing

## Abstract

The enzyme Dicer is a component of many small RNA (sRNA) pathways involved in RNA processing for post-transcriptional regulation, anti-viral response and control of transposable elements. Cleavage of double-stranded RNA by Dicer produces a signature overhanging sequence at the 3’ end of the sRNA sequence relative to a complementary passenger strand in a RNA duplex. There is a need for reliable tools to computationally search for Dicer cleavage signatures to help characterise families of sRNAs. This is increasingly important due to the rising popularity of sRNA sequencing, especially in non-model organisms. Here, we present stepRNA, a fast, local tool that identifies (i) overhang signatures strongly indicative of Dicer cleavage in RNA sequences, and (ii) the length of the passenger strand in sRNAs duplexes. We demonstrate the use of stepRNA with simulated and biological datasets to detect Dicer cleavage signatures in experimentally validated examples. Compared to currently available tools, stepRNA is more accurate, requires only sRNA sequence data rather than a reference genome, and provides information about other important features such as passenger strand length. stepRNA is freely available at https://github.com/Vicky-Hunt-Lab/stepRNA and is easily installable.

## 1 Introduction

Small RNAs (sRNAs) and their associated pathways are integral to post-transcriptional gene regulation, viral defence and transposon repression (Chapman and Carrington, 2007). In small-interfering RNA (siRNA) pathways, double-stranded RNA (dsRNA) sequences are cleaved into siRNA duplexes by enzymes (Bernstein et al., 2001). The siRNA duplex consists of a guide strand that will go on to become the mature siRNA which in most cases initiates degradation of a target transcript through complementary base pairing, and a passenger strand that will be degraded during maturation of the siRNA (Martinez et al., 2002; Matranga et al., 2005). Dicer, a RNase III family protein that is evolutionarily conserved across eukaryotes, including nematodes (Ketting et al., 2001; Gao et al., 2014), flies (Bernstein et al., 2001; Lee et al., 2004), plants (Henderson et al., 2006) and humans (Lee et al., 2013) is responsible for cleaving the duplexes of specific sRNA families.

**FIGURE 1:**
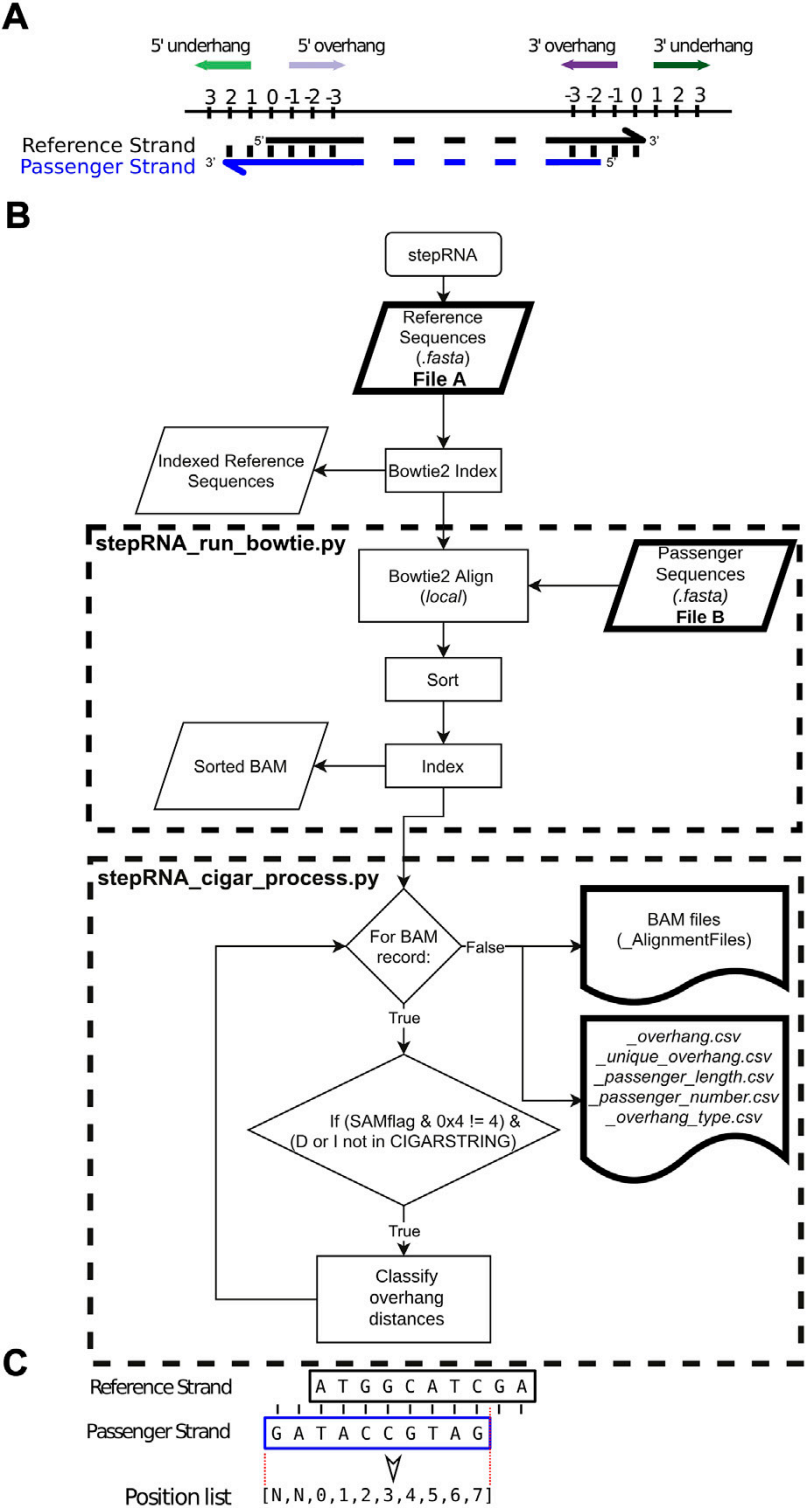
Overview of the stepRNA pipeline. **(A)** Diagram showing how stepRNA classifies a reference strand e.g. the guide RNA (black) and a passenger strand (blue) in a RNA duplex. A reference overhang (purple arrows) is represented by a negative score. A reference underhang (green arrows) is represented by a positive score. A blunt end is represented by a score of 0. In this example the reference strand has a 3′ overhang of 2 nt and a 5′ underhang of 2 nt. **(B)** Flowchart for the stepRNA pipeline showing an overview of the key steps. Modules that can be run independently are in dashed boxes. **(C)** Example of a siRNA duplex and the 0-based coordinate list, created by PYSAM, that stepRNA uses to classify the overhang distances for each siRNA duplex. The reference strand and passenger strand are represented by black and blue boxes, respectively. The dotted red line shows the region that is extracted, with softclipped bases in the passenger strand being represented as None (N).

Cleavage of dsRNA by the Dicer enzyme typically produces a 2-3 nucleotide (nt) overhang at the 3′ end of the RNA duplex (Figure 1), and a blunt end or 1–3 nt overhang at the 5′ end depending on the species and sRNA family being processed (Bernstein et al., 2001; Lee et al., 2004; Blumenfeld and Jose, 2016; Elvira-Matelot et al., 2016; Feng et al., 2020). For siRNAs, following Dicer cleavage the sRNA duplex, with its characteristic overhangs, associates with a pathway-specific Argonaute protein forming a RNA-induced silencing complex and degrades the passenger strand (Matranga et al., 2005). The mature RNA-induced silencing complex then identifies a target transcript based on sequence complementarity between the sRNA sequence and target RNA sequence, leading to degradation of the target RNA transcript or inhibition of translation (Vermeulen et al., 2005). For example, in *Caenorhabditis elegans*, cleavage of dsRNA by Dicer generates a 3 nt overhanging signature at the 3′end of the sRNA in the siRNA duplex. In *C. elegans*, the guide strand is 26 nt long and has a 5′ guanine (hereafter called 26G siRNA). The mature 26G siRNAs are involved in the ERGO-1 and ALG-3/4 siRNA pathways after the degradation of the passenger strand in the sRNA duplex. This role of 26Gs has been confirmed both *in vivo* and *in vitro* (Ma et al., 2004; Fischer et al., 2011; Blumenfeld and Jose, 2016). Dicer cleavage has also been well studied in other animal model organisms such as *Drosophila melanogaster,* whereby dsRNA is processed to produce 21–23 nt siRNA fragments containing a 2 nt overhang at the 3′ end of the guide siRNA. When mature, the 21–23 nt siRNAs result in targeted RNA degradation (Elbashir et al., 2001). In both *C. elegans* and *D. melanogaster*, dsRNA processing can begin from either end of both blunt ended sequences or sequences with short overhanging regions (Elbashir et al., 2001; Blumenfeld and Jose, 2016). Human Dicer proteins generate duplexes of sequences that are 21–23 nt containing a 2 nt 3′overhang from 500bp dsRNA substrates (Provost et al., 2002). Dicer is also essential in viral defence where it cleaves viral RNA. For example, Dicer has been shown to cleave viral RNA leaving a 2 nt 3′ overhang in dsRNA/sRNA viruses infecting *D. melanogaster* (Antoniewski, 2014). Collectively, Dicer plays an important role in sRNA pathways and viral defence across a diverse range of species.

In the plant model organism *Arabidopsis thaliana*, RNA-Directed DNA Methylation (RdDM) is the unique mechanism in plants for silencing transposable elements (TEs) and genes by sRNAs (Erdmann and Picard, 2020). There are four Dicer-like proteins in *A. thaliana* (Xie et al., 2004) involved in the sRNA pathway required for RdDM. The *de novo* RdDM, similar to the RNAi pathway in small RNA cleavage and biogenesis (Markulin et al., 2021), utilises 21 and 22 nt siRNAs cleaved by Dicer-like 4 and 2 respectively to establish DNA methylation at novel loci e.g. upon a novel TE insertion or after reactivation of a TE (Henderson et al., 2006; Markulin et al., 2021). The methylation is then maintained by a separate RdDM pathway that uses 24 nt siRNAs, cleaved by Dicer-like 3 (Henderson et al., 2006). Maintenance of RdDM by 24 nt siRNAs is the best characterised small RNA pathway in *A. thaliana* and the most abundant siRNA species present (Meyer et al., 2015; Erdmann and Picard, 2020).

Characterisation of sRNA families has become increasingly important due to the rising popularity and capability of sRNA sequencing in non-model organisms and the subsequent identification of previously unrecognised siRNAs (Bernhardt et al., 2012; Moser et al., 2013; Suleiman et al., 2022). A key goal in siRNA biology is to characterise sequences into families most likely to belong to the same pathway and therefore have similar RNA targets. There are several features that can be used together to characterise the sRNAs in a specific pathway. For example, a specific Argonaute or set of Argonaute proteins involved in guiding the siRNA to its target sequence is specific to a sRNA pathway and the associated classes of siRNA. In addition, the length of a mature siRNA sequence, the first 5′ starting base, and the siRNA processing mechanism are key features that can also be used to characterise and classify siRNA families. Identification of Dicer cleavage signatures is critical in this classification process. Currently available sRNA bioinformatic analytical tools characterise sRNAs sequence length or the first 5′ starting base, but most do not analyse Dicer cleavage signatures (e.g. Gebert, Hewel and Rosenkranz, 2017; Pogorelcnik et al., 2018). To our knowledge, only one tool (signature.py) has been reported in the literature to specifically identify Dicer cleavage signatures in RNA sequences (Antoniewski, 2014). This tool relies on the availability of a reference genome to align the sRNA reads to and does not provide information about the passenger strand or the 5′ end of the siRNA duplex. There are two main limitations of relying on the coordinates of RNA *loci* in genomic data to uncover Dicer cleavage signatures: (i) this method cannot be used for non-model organisms where genome assemblies are unavailable or are of poor quality, and (ii) the siRNAs derived from sequences that originate from spliced precursor sequences, such as sequences that span exon-exon junctions of mature mRNAs (Ruby et al., 2006; Han et al., 2009; Gent et al., 2010) cannot be readily detected. Identification of Dicer cleavage signatures independent of a reference genome is essential to characterise sRNA for diverse organisms and classes of siRNA. Here, we present stepRNA, a fast, local alignment-based tool for the automated discovery of Dicer cleavage signatures in sRNA datasets. stepRNA works independently of a genome sequence and identifies (i) the number and length of overhanging sequences at the 5′ and 3′ ends and (ii) the length of passenger sequences. stepRNA outputs are user-friendly and easily adapted to make figures and for downstream analyses.

## 2 Material and methods

### 2.1 stepRNA implementation, description and output

#### 2.1.1 Implementation

stepRNA is implemented in python3 and can be easily installed using pip (*pip install stepRNA*). It has been tested in a Unix OS environment and requires PYSAM (v0.16), biopython (v1.78) and numpy (v1.19). Detailed installation instructions and the manual can be found on the GitHub page (https://github.com/Vicky-Hunt-Lab/stepRNA).

#### 2.1.2 Input files

stepRNA requires two adapter-trimmed FASTA files from sRNA-sequencing data as input (1) reference sequences, e.g. the guide siRNAs of interest, hereafter called File A, and (2) potential passenger sequences, hereafter called File B. For example, File A could comprise sequences hypothesised to be Dicer cleaved, such as 26G siRNAs in *C. elegans*, if the user is interested in looking at a specific predetermined class of siRNAs or sequences of various lengths with unknown Dicer processing. File B sequences should comprise any potential passenger sequences, for example, all sequences between 18 and 30 nt in length. File A and B require unique headers made by the user or can be generated by stepRNA using the flag *-u.*

#### 2.1.3 Read alignment

A BOWTIE2 index is built from File A; File B reads are then aligned to this index using BOWTIE2 without allowing any mismatches between the File A and File B reads (Langmead and Salzberg, 2012). This generates candidate siRNA duplexes that must have a minimum number of bases (default: shortest query sequence length from File B) with a perfect reverse complement between the reference read in File A, and query read in File B. BOWTIE2 was run in the *local alignment* mode which allowed softclipping at the end of the aligned reads. This retains information about the distances between the end of the reference and query strands. Aligned reads are output as an indexed BAM file (Figure 1B).

#### 2.1.4 CIGAR string processing

The overhang distance, i.e. the number of nucleotides that a reference sRNA read from File A extends beyond a query sRNA read from File B, or underhang distance, i.e. the number of nucleotides that a reference sRNA read from File B extends beyond a query read from File A are then calculated (Figure 1A). stepRNA uses CIGAR string information for each successfully aligned duplex using PYSAM (https://github.com/pysam-developers/pysam). Briefly, for each duplex in the BAM file, an aligned query read (from File B) is extracted as a 0-based integer list representing the coordinates where the sequence has aligned to a single reference sequence from File A (Figure 1C). This enables stepRNA to identify an overhang or underhang and the length at the 5′ and 3′ end by comparing the first (5′ end) and last (3′ end) values in the python list, respectively. The distance between the end of the reference sequence and the passenger sequence can then be calculated using PYSAM functions (summarised in Table 1). Importantly, stepRNA identifies if the query sequence (from File B) overhangs, underhangs or is blunt ended relative to both ends of the reference read (from File A). In the output, an overhang distance is represented by a negative integer and an underhang distance by a positive integer relative to the reference strand (Figure 1A).

**TABLE 1:** The stepRNA methodology for calculating the distance between a reference strand and the passenger strand.

Type	siRNA duplex example	5′ end	3′ end	Coordinate List [ …,…,…,E]	Distance Calculation
		Coordinate List [S,…,…, … ]<	Distance Calculation		
Exact	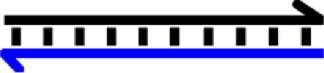	If S is 0	0	If E is (reflen - 1)	0
Overhang	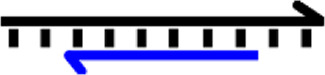	If S > 0	qs	If E < (reflen - 1)	E—(reflen - 1)
Underhang	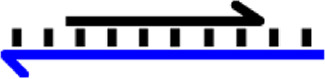	If S is N	- A	If E is N	passlen—qe

qs = *query_alignment_start* position is the base pair position along the reference where the query alignment starts (from PYSAM), qe = *query_alignment_end* position is the base pair position along the reference where the query alignment ends (from PYSAM), reflen = reference sequence length, passlen = passenger sequence length, the arrow on the end of the examples represents the 3′ RNA end. S represents the 5′ end reference value in the position list. E represents the 3′ end reference value in the position list. N represents ‘None’. The black strand is the reference strand from file A; The blue strand is the query strand from file B.

#### 2.1.5 Overhang length enrichment

For the 5′ and 3′ overhang counts of the reference sequence, i.e. the number of reference sequences that align to a passenger sequence, a log-odds value was calculated by obtaining the log of the ratio for an overhang count and the mean count at the ends. Z-scores were calculated using the Wald test (Molenberghs and Verbeke, 2007).

#### 2.1.6 Output files

stepRNA generates five summary files:

1) Counts of the number of duplexes for each underhang or overhang distance where a reference can be represented multiple times, i.e. representing expression data (suffix: *overhang.csv*).
2) Counts of the number of duplexes for each underhang or overhang distance where only unique reference sequences are counted (suffix: *unique_overhang.csv).*
3) The number of passengers for each reference sequence (suffix: *passenger_number.csv*).
4) The passenger length (suffix: *passenger_length.csv*).
5) A summary of the number of overhangs and underhangs (suffix: *overhang_type.csv*).

Example output files can be found at https://github.com/Vicky-Hunt-Lab/stepRNA/example_data/example_output/. The reference reads (from File A) with a matching query read (from File B) passenger are also stored in BAM files according to the 5′ or 3′ end distance classification. This allows a more detailed analysis of specific overhang or underhang lengths. The modularised stepRNA pipleine allows the CIGAR string processing to be run on individual overhang or underhang classifications using the corresponding BAM file (*stepRNA_cigar_process.py*).

### 2.2 Simulated data generation

In order to test our algorithm, simulated reference and query sequences with known overhang and underhang distances were generated (Dataset A, script and data available at https://github.com/Vicky-Hunt-Lab/stepRNA/example_data). From 20,000 21 nt randomly generated reads a subset of 797 sequences were randomly selected to be sRNAs which form duplexes i.e. potentially cleaved by Dicer, and these were used as the query reference reads (‘File A’, Supplementary Table S1). Passenger strands with known overhangs and underhangs were created for the 797 sequences. To simulate a real data set 49% of the duplexes multimapped to the genome to better represent a real sRNA dataset (Supplementary Table S1). These reads together with the 19,203 reads without duplexes (representing background noise) were used as input for File B. For comparison to signature.py, a simulated reference genome was also created by combining reference and passenger sequences, with five random nucleotides separating each sequence to allow an alignment of the simulated reference and query sequences. Overhangs and underhangs were filled in with complementary bases to allow valid alignments to the genome.

### 2.3 Biological data

#### 2.3.1 *C. elegans*

A phosphate-independent sRNA sequencing library from a *C. elegans* embryo (GEO: GSM801363; Fischer et al., 2011) was used to validate stepRNA with a biological dataset. Adapters were trimmed using CUTADAPT v1.18 (Martin, 2011) and converted to FASTA files. 26G and 22G are experimentally confirmed mature siRNAs, therefore 2 reference files (File A) were produced, one for either 26G or 22G sRNAs. A file containing query sequences (File B), was generated by filtering sRNA reads between 15 nt to 30 nt. Read files were collapsed using NGS toolbox *collapse* (Rosenkranz et al., 2015) to speed-up overhang detection.

#### 2.3.2 *A. thaliana*

Two *A. thaliana* sRNA libraries, a wild-type and a *dcl2dcl3dcl4* triple mutant in Columbia background (GEO: GSM1845210 and GSM1845222; Elvira-Matelot et al., 2016) were obtained from the Arabidopsis Small RNA Database (Feng et al., 2020). These had been previously adapter trimmed and length filtered to 18–28 nt. All the *A. thaliana* miRNAs detected using the miRBase release 22.1 (Kozomara et al., 2019) were removed before collapsing the sRNA sequences using NGStoolbox *collapse* (Rosenkranz et al., 2015). For the *A. thaliana* analysis, the same set of 18–28 nt sequences was used for files A and B to search for all possible Dicer processed sequences, rather than a specific class, e.g. 26Gs analysed in the *C. elegans* dataset. After inspecting the stepRNA output, the WT and DCL sRNA sequence data was also filtered to retain sRNAs that were 24 nt long to be used as File A input. After the stepRNA procedure, the sRNA read count data were summarised using count information generated by NGStoolbox *collapse* (Rosenkranz et al., 2015), which is retained in the FASTA file when using the -u flag. Expression data was then normalised using reads per million (RPM).

### 2.4 Spike-in data generation for single- and multiple-sequence spike-in analyses

Spike-in datasets were generated using custom algorithms (https://github.com/Vicky-Hunt-Lab/stepRNA/example_data/makeSpike.py). Two sets of spike-in data were generated to test stepRNAs ability to detect overhangs in *C. elegans* biological datasets: i) A single siRNA was selected randomly and multiple ‘non-collapsed’ siRNA reference reads of the same sequence (representing expression data). Spike-in data for the non-collapsed reference siRNAs was generated from a randomly selected 26G or 22G reference read that had a passenger in the biological data. Each single read was then used as a template to generate 5,000 identical passenger strands for both 26G and 22G, with the following overhangs: the 26G passengers have a blunt 5′end and a 1 nt overhang at the 3′end; and the 22G passengers have 2 nt overhangs at both ends or ii) File A containing only ‘collapsed’ unique guide siRNA reads (representing a set of unique sequences belonging to a class of siRNA). To generate spike-in data for the collapsed reference siRNAs, 5,000 randomly generated unique 24 nt reference sequences, beginning with either a cytosine (24C) or adenine (24A) were created. Passenger strands were then generated with a 1 nt 5′ overhang and a 2 nt 3′ overhang for each generated 24 nt reference read. The spike-in sRNAs and passengers were combined with the respective reference (File A) and query (File B) FASTA files for the biological datasets.

### 2.5 Running stepRNA

Description of stepRNA’s algorithm is detailed above. stepRNA can be run on the command line following the general example:

stepRNA -r./path/to/FILE_A.fa -q./path/to/FILE_B.fa -n PREFIX -d./path/to/OutputDirectory

stepRNA was run using default settings unless otherwise stated, with the siRNA reference and passenger files input using *-r* and *-q* respectively.

stepRNA is set to have a conservative, no mismatches approach to identifying sRNA duplexes. However, if the user wishes, they could adapt the Bowtie2 command in *stepRNA/stepRNA_run_bowtie.py* (line 20 in Supp Methods) to allow mismatches depending on their specific requirements by changing the *--ma* (match bonus), *--mp* (match penalty) and *--score-min,* values. For example, if the user wanted to allow one mismatch then they could change these values to *--ma 1, --mp 0,0 --score-min -L,-1,1*.

### 2.6 Running signature.py

Signature.py (Antoniewski, 2014) was tested on simulated siRNA data (Section 2.2) and two biological data sets for *C. elegans* or *A. thaliana* (Section 2.3) for comparison to stepRNA. For signature.py, File B sequences were aligned to the genome of interest using Bowtie (Langmead et al., 2009), as recommended by the authors (Antoniewski, 2014). The output BAM file was then input into signature.py, and the output table was adapted to allow a direct comparison to stepRNA because signature.py calculates overlapping distances compared to stepRNA, which calculates overhang and underhang distances relative to the reference sequence. For simulated reads, the number of duplexes with a calculated underhang and overhang distance were plotted, and for biological and spike-in datasets, the percentage of the total reference reads were plotted. All plots were generated using R (R core Team, 2021).

Z-scores were taken from the signature.py output. Percentage plots and z-score plots were plotted using R.

## 3 Results

### 3.1 stepRNA accurately identifies overhang lengths in simulated sRNA datasets

**FIGURE 2:**
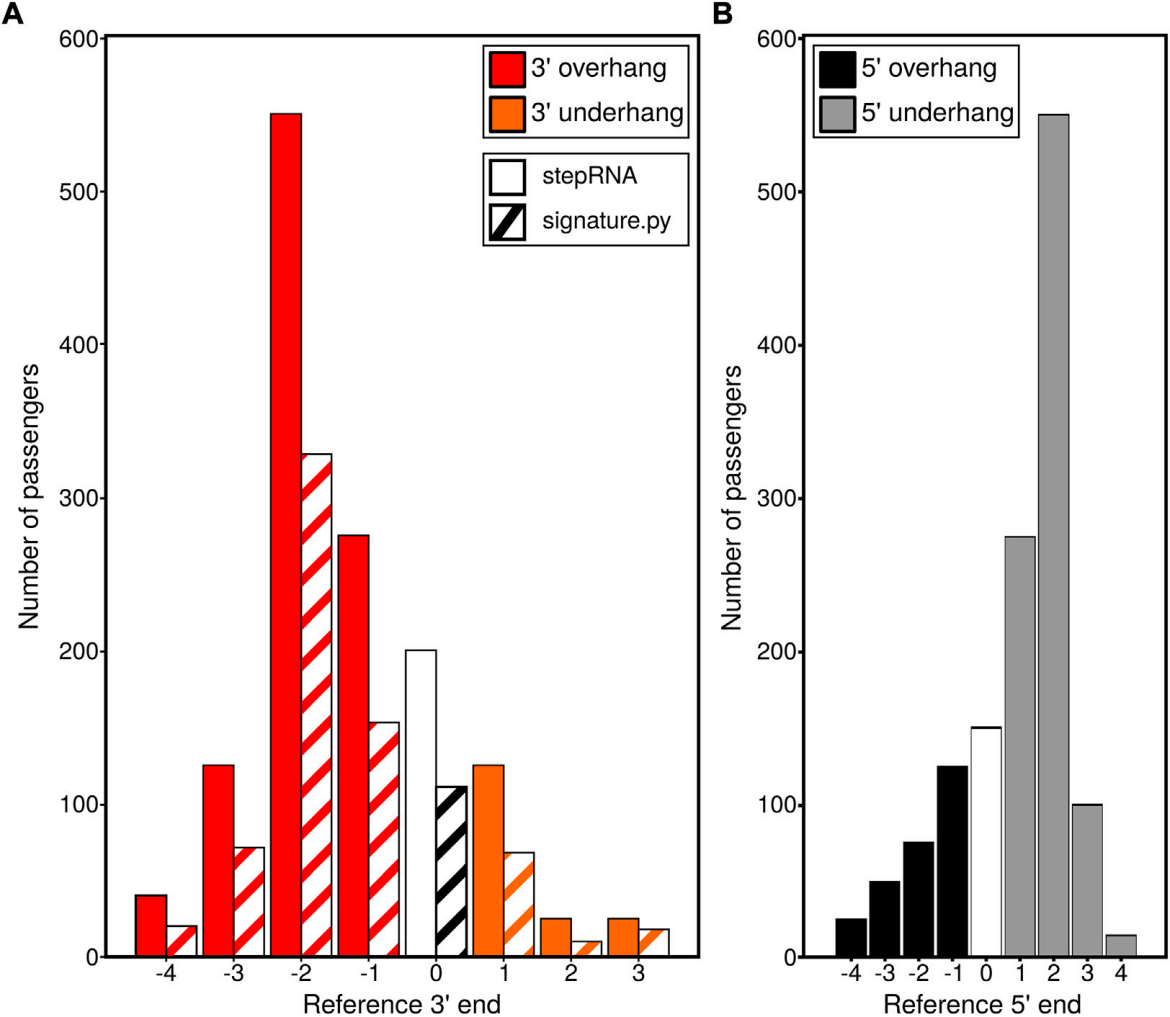
stepRNA outperforms the currently available tool signature.py on a simulated dataset. Simulated data were generated for File A (the reference sequence e.g. the predicted guide strand) and File B (the passenger strand) with known lengths, passenger numbers and overhang or underhang lengths. These data were run with stepRNA and signature.py. **(A)** Bar graphs show the number of predicted passenger strands for different overhang and underhang distances, identified by stepRNA (solid bars) and signature.py (hashed bars) at the 3′ reference end. An overhang by the reference strand is represented by a red bar, a reference strand underhang by an orange bar and a blunt end is represented by a white or black and white bar. **(B)** Bar graphs showing the 5′ reference end counts for the distances identified by stepRNA n.b. signature.py only provides 3′ reference end information and there is no data for the 5′ end. A reference overhang is represented by black bars, a reference underhang by grey bars and a blunt end is represented by a white bar.

A simulated sRNA-seq dataset containing sRNA duplexes with known overhang and underhang distances was used to compare stepRNA and signature.py (Antoniewski, 2014). Investigation of the 3′ end of the sRNA in duplexes revealed stepRNA correctly identified all the overhangs and underhangs in the simulated dataset, compared with signature.py which only identified 59% of the duplexes correctly (Figure 2A, Table 2). The improved classification by stepRNA is due to the direct alignment of sRNA reads to one another, which accounts for all possible combinations of reads *cf.* signature.py which aligns reads to the genome and uses single *loci* coordinates i.e. missing information about multimapping reads. Next, we investigated the 5′ end of the simulated sRNA duplexes. stepRNA could classify the 5′ end of reads from the simulated dataset with 100% accuracy (Figure 2B). However, signature.py does not have this function and cannot identify overhangs and underhangs at the 5′ end. The additional information about the 5′ end is beneficial because it can be readily compared against known Dicer cleavage signatures for further confidence in the results and can provide more information about newly identified sRNAs. For example, in *C. elegans,* the Dicer cleavage signature is a 5′ 4 nt overhang and a 3′ 3 nt overhang (Blumenfeld and Jose, 2016). Our results demonstrate that stepRNA can accurately detect overhangs and underhangs at both ends of sRNA duplexes.

**TABLE 2:** stepRNA detects more sRNA duplexes correctly compared to signature.py at the 3’ end of sRNA duplexes.

Overhang distance at the 3′ end (nt)	Expected Number of sRNA Duplexes	sRNA Duplexes found by stepRNA	sRNA duplexes found by signature.py*
−4	40	40	20 (50%)
−3	125	125	69 (52%)
−2	550	550	328 (60%)
−1	275	275	153 (56%)
0	200	200	111 (55%)
1	125	125	68 (54%)
2	25	25	10 (40%)
3	25	25	18 (72%)
Total	1365	1365	777 (57%)

*Percentage of the expected number of duplexes in the simulated dataset are shown in brackets for signature.py

### 3.2 Dicer processing signatures can be identified in biological data sets

We have shown that stepRNA can correctly detect overhangs from simulated data. However, in biological datasets, non-Dicer cleaved sRNAs might hinder the detection of Dicer cleaved overhang signatures. We therefore tested stepRNA on published biological data from two highly diverged species to confirm that stepRNA could detect a Dicer cleavage signature using siRNA pathways characterised in *C. elegans* and *A. thialiana*. We also ran signature.py on the same two datasets for comparison.

#### 3.2.1 *C. elegans* 26G (Dicer cleaved) and 22G (Dicer independent) siRNAs

From a wild-type *C. elegans* sRNA sequencing library (Fischer et al., 2011), we extracted 26G and 22G sRNAs as reference reads (File A). The 26Gs siRNAs are Dicer cleaved (Blumenfeld and Jose, 2016), and 22Gs are produced by RNA-dependent RNA polymerase (RdRP) without Dicer (Fischer et al., 2011). Therefore, stepRNA should uncover evidence of a Dicer cleavage signature from the 26G but not 22G sequence data. We ran stepRNA for both collapsed and non-collapsed sequences in the reference files (File A). The collapsed file contains unique reads and represents each siRNA sequence once and can identify unique sRNA sequences that are Dicer cleaved. The non-collapsed file contains multiple copies of the same sequences and represents expression data where an identical sRNA sequence may be present multiple times. This can be used to identify the amount of Dicer cleavage occurring on the reference sequences of interest. Potential passenger strands included reads of multiple lengths (see Methods).

##### 3.2.1.1 Collapsed datasets

**FIGURE 3:**
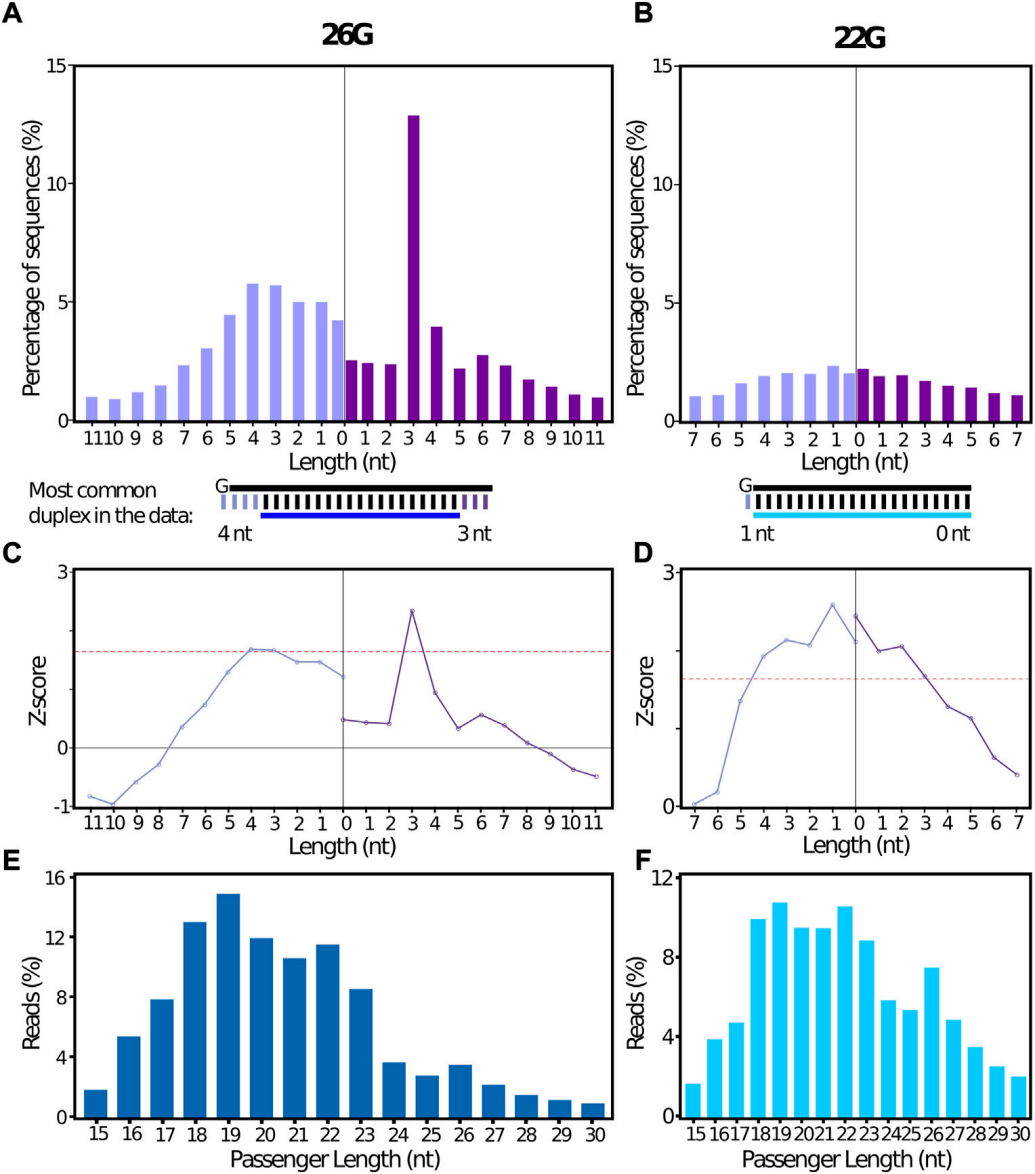
stepRNA identifies Dicer cleavage signatures from a biological data set containing *C. elegans* 26G siRNAs sequence data. stepRNA was run using a collapsed file of reference siRNAs reads i.e. that only contained unique sequences that are either **(A)** 26 nt long and start with a 5′ guanine (26Gs; n = 28,651) or **(B)** 22 nt long and start with a 5′ guanine (22Gs; n = 106,723) (File A) against potential passenger sequences which include all siRNAs that are 15–30 nt long (File B). Sequence data was taken from (Fischer et al., 2011) (GEO: GSE32366). Bar graphs show the percentage of reference reads with a 3′ overhang (dark purple) or 3′ underhang (light purple), for **(A)** 26Gs and **(B)** 22G siRNAs. The most common siRNA duplex for 26G and 22G, as predicted by stepRNA, is illustrated below the plot. Line graphs showing the z-scores for **(C)** 26G and **(D)** 22G. Red line indicates *p* = 0.05. Bar graphs show the predicted passenger length distribution produced by stepRNA for **(E)** 26G (dark blue) and **(F)** 22G siRNAs (light blue). In total, 22,003 and 49,059 passengers were predicted, for 26Gs and 22Gs respectively, by stepRNA.

A complementary passenger sequence was identified by stepRNA for 23.5%, and 12.2% of the 26G and 22G collapsed reference sRNA reads, respectively (Supplementary Table S2A). For the 26G sequences, a distinct 3′ 3 nt overhang peak was observed for the sRNA relative to the predicted passenger strand (Figure 3A) and this Dicer cleavage signature was confirmed by a Z-score value calculated by stepRNA to be above our cut-off of 1.645 (equivalent to a significance at *p* = 0.05). This indicates that stepRNA has correctly identified a Dicer cleavage signature for *C. elegans* 26G siRNAs (Figure 3C, Supplementary Table S2B). As expected, no distinct overhang length at the 5′ or 3′ end of the duplex was identified for the 22G sequences confirming there is no evidence of Dicer cleavage (Figures 3B,D, Supplementary Table S2C). Enrichment of a particular underhang length was also not observed for 22Gs or 26Gs (Supplementary Figure S1, Supplementary Table S2B and C). Our results are consistent with previous findings by Blumenfeld and Jose (2016) and Fischer *et al.* (2011). stepRNA also reports the predicted passenger strand length distribution. The most frequent passenger length was 19 nt for both 26G and 22G siRNAs (Figures 3E,F, Supplementary Table S2D). The majority of predicted passenger lengths for 26G were 18—22 nt. This is consistent with 19–22 nt passenger lengths observed *in vivo* and *in vitro* (Blumenfeld and Jose, 2016). Consistent with the most common passenger lengths, a significant 3—4 nt overhang enrichment was also observed at the 5′ end in the 26G RNAs (Figures 3A,C). Together these results support that stepRNA correctly identifies Dicer cleaved siRNA duplexes from *C. elegans* sRNA datasets.

##### 3.2.1.2 Non-collapsed datasets

A Dicer cleavage signature was also detected using datasets that had not been collapsed. A passenger strand sequence was only predicted for 3.3% of non-collapsed 26G siRNAs (Supplementary Table S2A), but crucially, stepRNA was still able to detect a Dicer cleavage signature at the 3′ end (Supplementary Figure S2A and C, Supplementary Table S2E). Consistent with collapsed data analysis, the 22G siRNAs had no Dicer cleavage signature (Supplementary Figure S2B and D, Supplementary Table S2F). These results demonstrate that stepRNA can identify Dicer cleavage signatures in expression data.

#### 3.2.2 Identification of *A. thaliana* 24 nt dicer cleaved siRNAs in wild type and *dcl2dcl3dcl4* mutant

In *A. thaliana,* 24 nt siRNAs are involved in the RdDM silencing of TEs and genes (Erdmann and Picard, 2020). These siRNAs form Dicer cleaved sRNA duplexes with other 24 nt sequences with a signature overhang of 2 nt at the 3′ end (Henderson et al., 2006; Meyer et al., 2015; Singh et al., 2019). We ran stepRNA on previously published *A. thaliana* sRNA wild type and *dcl2dcl3dcl4* mutant sequence data between 18 and 28 nt in length (Elvira-Matelot et al., 2016) to search for Dicer cleavage signatures and test how stepRNA identifies Dicer cleaved sRNAs in mixed or raw sRNA sequence data, i.e. where the sRNA length or first base is unknown.

**FIGURE 4:**
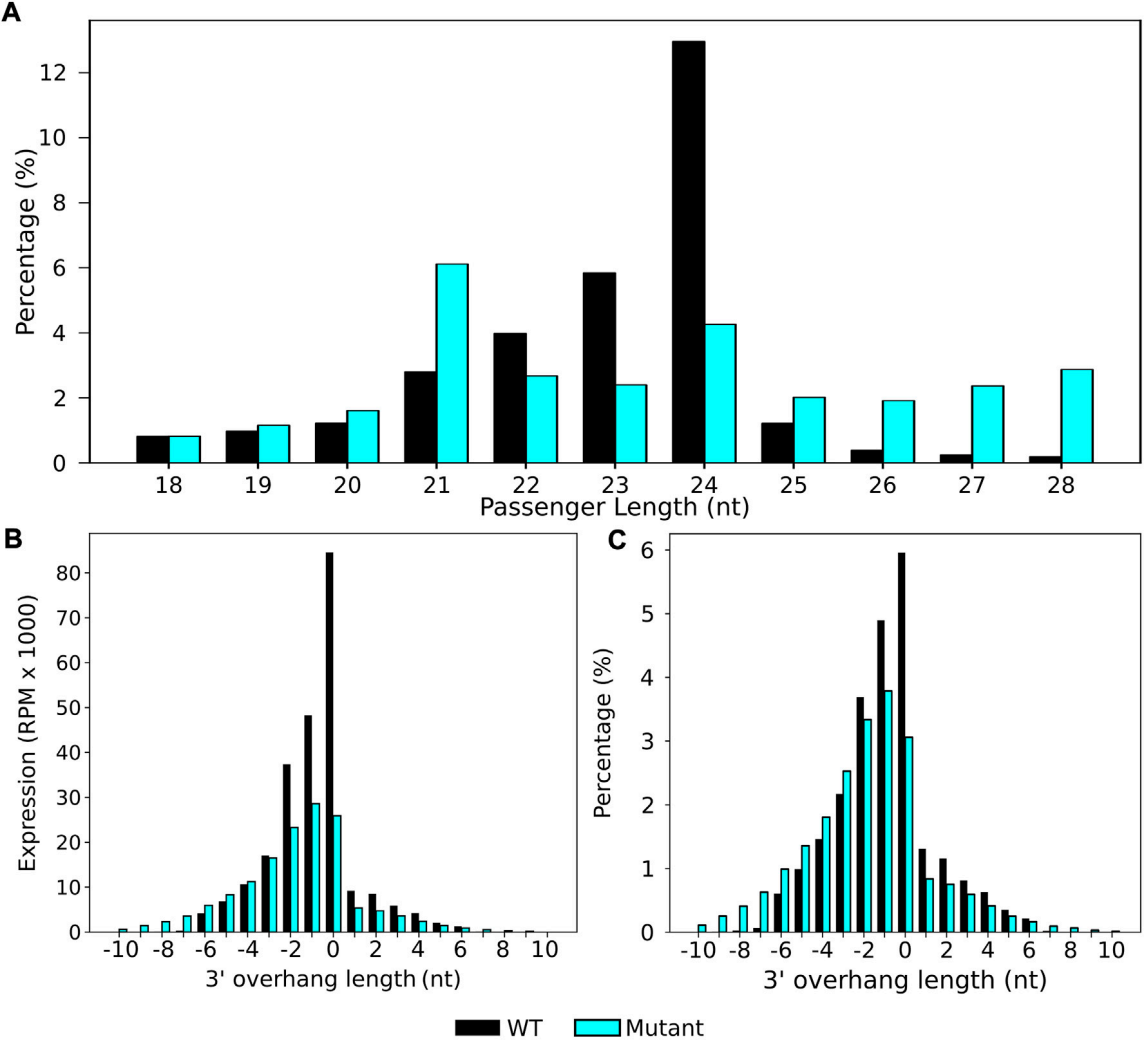
Dicer cleavage signatures for 24 nt siRNAs were uncovered in a wild-type *A. thaliana* sRNA raw data by stepRNA. stepRNA was first run using a collapsed file of reference sRNAs reads *i.e.* that only contained unique sequences, as both the reference and query input, from a wild-type (black) and *dcl2dcl3dcl4* mutant (blue) *A. thaliana* sRNA library. **(A)** Bar graphs show the length distribution of sRNA sequences predicted to have a passenger strand n. b. the data includes both the predicted sRNA and its passenger strand because these cannot be distinguished at this stage. stepRNA output for both wild-type and *dcl2dcl3dcl4* mutant strains are shown, identifying that 24 nt sRNAs are most likely to have a passenger strand in wild type but not *dcl2dcl3dcl4* mutant. **(B)** Bar graph showing the expression of sRNA in duplexes and the 3′ overhang length in the predicted sRNA duplex. **(C)** Bar graph showing the percentage of sRNA duplexes with at least one overhang of the corresponding length at the 3′ end (n = 2908404 WT, n = 2733702 *dcl2dcl3dcl4* mutant).

stepRNA identified that 24 nt sRNAs were the most common sRNAs that have a predicted passenger strand in wild type, but not in the sRNA sequence data of the *dcl2dcl3dcl4* mutant (Figure 4A), supporting previous findings that 24 nt sRNAs are Dicer cleaved in *A. thaliana* (Henderson et al., 2006; Meyer et al., 2015)*.* stepRNA was used to identify if there was an overhang in the sRNA duplex and the length of the overhanging sequence. Most commonly, duplexes were predicted to be either blunt-ended (overhang of 0 nt) or have a 1–2 nt 3′ overhang at the 3′ end of the sRNA. These cleavage signatures were not found in the *dcl2dcl3dcl4* mutant (Figure 4B, C, Supplementary Table S3A and C).

**FIGURE 5:**
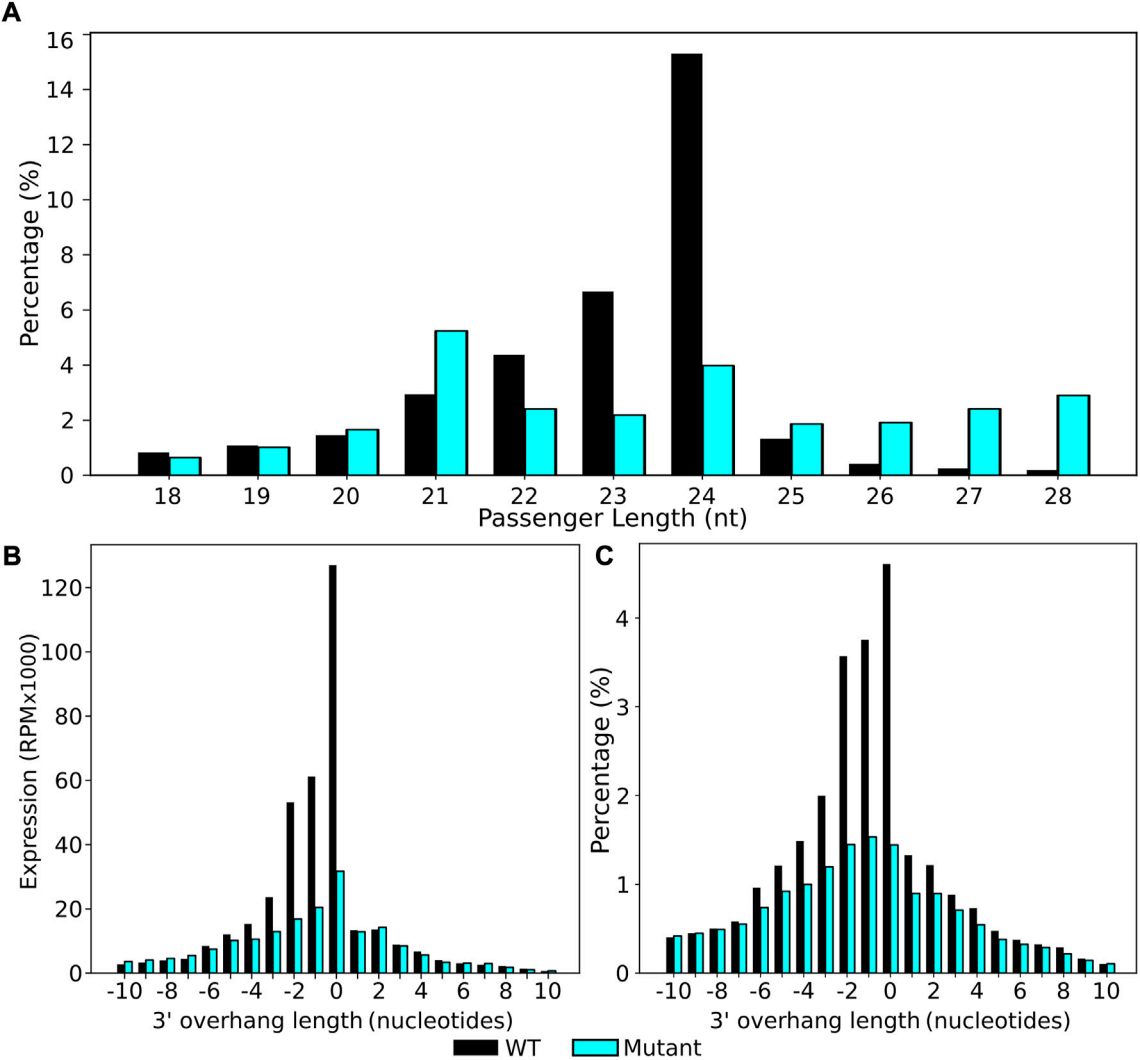
stepRNA predicts that *A. thaliana* 24 nt sRNAs form duplexes with other 24 nt sequences with a 3′ Dicer cleavage signature. 24nt sRNA reads were extracted from raw data and analysed with stepRNA. All raw reads were used as potential passenger strands to identify the most likely passenger strand length and Dicer cleavage signatures. Bar graphs showing **(A)** the predicted passenger strand lengths of wild-type (black) and *dcl2dcl3dcl4* mutant (blue) strains, **(B)** sRNA expression of sRNAs in duplexes and the 3′ overhang length in the predicted sRNA duplex, **(C)** the percentage of sRNA duplexes with at least one overhang of corresponding length overhang at the 3′ end (n = 2908404 WT, n = 2733702 *dcl2dcl3dcl4* mutant).

To investigate this further, we extracted all 24 nt sRNAs from the data set and used these as our reference sRNA of interest (File A). stepRNA was re-run with 24 nt (File A) against all 18–28 nt sRNAs (File B) to specifically identify the most common passenger strand length of 24 nt sRNAs (Figure 5, Supplementary Table S4). In agreeance with previously published studies (Singh et al., 2019) we found that 24 nt was also the most common passenger strand, confirming that 24 nt sRNAs most commonly form a duplex with other 24 nt passenger sequences (Figure 5A, Supplementary Table S4A and B). When using 24 nt sRNAs as a reference input to stepRNA, we confirmed that the expected enrichment of 1–2 nt overhang was present in the wild type sRNA dataset with an additional unexpected blunt ended 0 nt present (Figure 5B, C; Supplementary Figure S3); n.b. it is not possible to identify which strand is the guide sRNA strand and which is the passenger strand because they are both the same length). There was only low detection of an overhang in the *dcl2dcl3dcl4* mutant, as expected, because the Dicer protein that cleaves 24 nt sRNAs is defective in the mutant strain (Figure 5B, C, Supplementary Table S4C and D). In summary, stepRNA was able to identify 24 nt sRNAs that are Dicer cleaved and that they most commonly have 24 nt passenger strands in sRNA duplexes. Furthermore, these results demonstrate that stepRNA can be used to identify Dicer cleavage signatures in mixed raw sequence data where information about the Dicer cleaved sRNA is unknown.

##### 3.2.2.1 Comparison with signature.py

**FIGURE 6:**
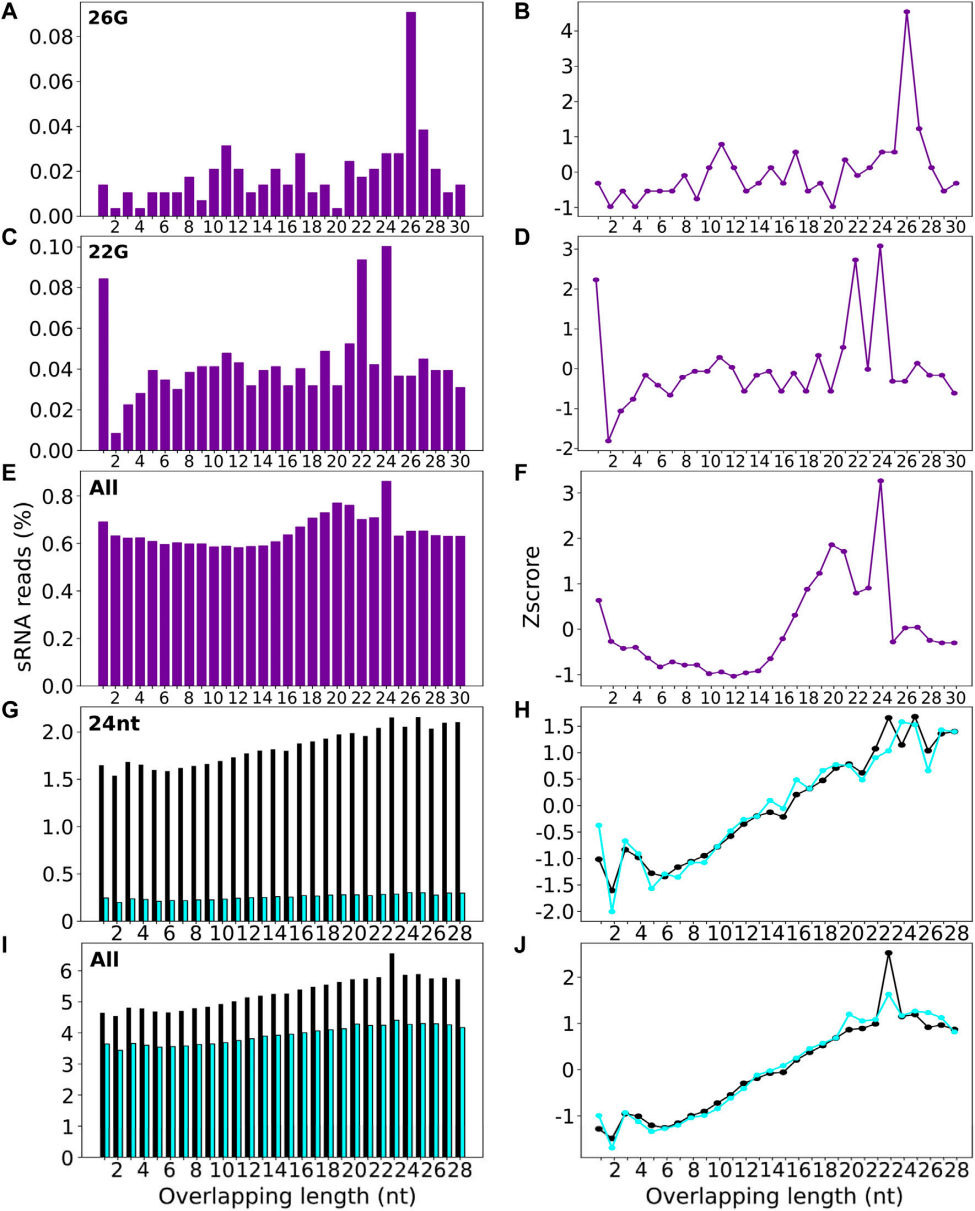
Signature.py detects low percentage of overlapping sRNA sequences in real datasets. signature.py was run on 26G sRNAs **(A and B)**, 22G sRNAs **(C and D)** and all sRNAs **(E and F)** for *C. elegans,* and 24 nt sRNAs **(G and H)** (purple), and all sRNAs **(I and J)** for WT (black) and *dcl2dcl3dcl4* mutant cell lines (blue) for *A. thaliana.* Output barplots (left) show the length of overlapping sequence identified for sRNAs aligned in a potential duplex. Lineplots (right) show the enrichment z-score for the overlapping lengths shown in the boxplots.

Signature.py was run on all *C. elegans* sRNA reads and datasets filtered for either 26G or 22G sequences (Supplementary Table S5 and 6, Figure 6). The input for signature.py is a genome file and a single sRNA file, and it doesn’t enable selection of a subset of sRNAs of interest e.g. of a particular length. Using a single sRNA length input limits signature.py to only uncover duplexes with the same sRNA length, which is problematic if the guide and passenger sRNAs have different lengths i.e. when using the 26G sRNA file as input, only duplexes with other 26Gs can be identified. This means that signature.py is unable to detect any duplexes that are Dicer signature because it is lacking the sequence information about the true passengers, which are predominantly 19 nt in length. A comparative analysis of 26G sRNAs found that stepRNA identified 12.8% of sequences that had the 3 nt 3′ overhang Dicer cleavage signature, compared to 0.02% of sequences with a 3 nt 3′ overhang (represented as an overlap of 23 i.e. 26—3) identified by signature.py (Supplementary Table S5, Figure 6). We also ran signature.py with all sRNAs reads to assess if this tool could identify the expected Dicer signature for 26Gs. Signature.py identified that overlapping sequence regions of 24 nt (0.086% of all reads) were most common. This is in contrast to the results generated by stepRNA (Figure 3) and experimental data (Blumenfeld and Jose, 2016) which implies that the 26Gs are Dicer processed and have a 3′ overhang of 3 nt and an overlapping sequence of 19 nt, not 24 nt. The passenger strand of 22G are predominantly 22Gs and a comparison between stepRNA and signature.py can be made. Both tools did not detect a Dicer cleavage signature for 22Gs, as expected (Supplementary Table S6, Figure 3, Figure 6). Signature.py was also run on the same *A. thaliana* dataset as stepRNA, including all sRNAs and for a dataset of 24-nt filtered sequences. Overall, approximately eight times the number of overlapping signatures were identified in wild type compared with *dcl2dcl3dcl4* mutant sequence data, suggesting that signature.py can identify duplexes that are Dicer cleaved. However, there was no distinct pattern identifying a particular overlapping length (Figure 6, Supplementary Table S7 and S8). This contrasts with stepRNA and experimental data (Henderson et al., 2006; Meyer et al., 2015; Erdmann and Picard, 2020), which identified that 24 nt passengers and a 0–2 3′ overhang was most commonly associated with Dicer cleaved sRNAs (Figures 4, Figure 5).

### 3.3 *In silico* spike-ins reveal stepRNA confidently recovers overhang information

**FIGURE 7:**
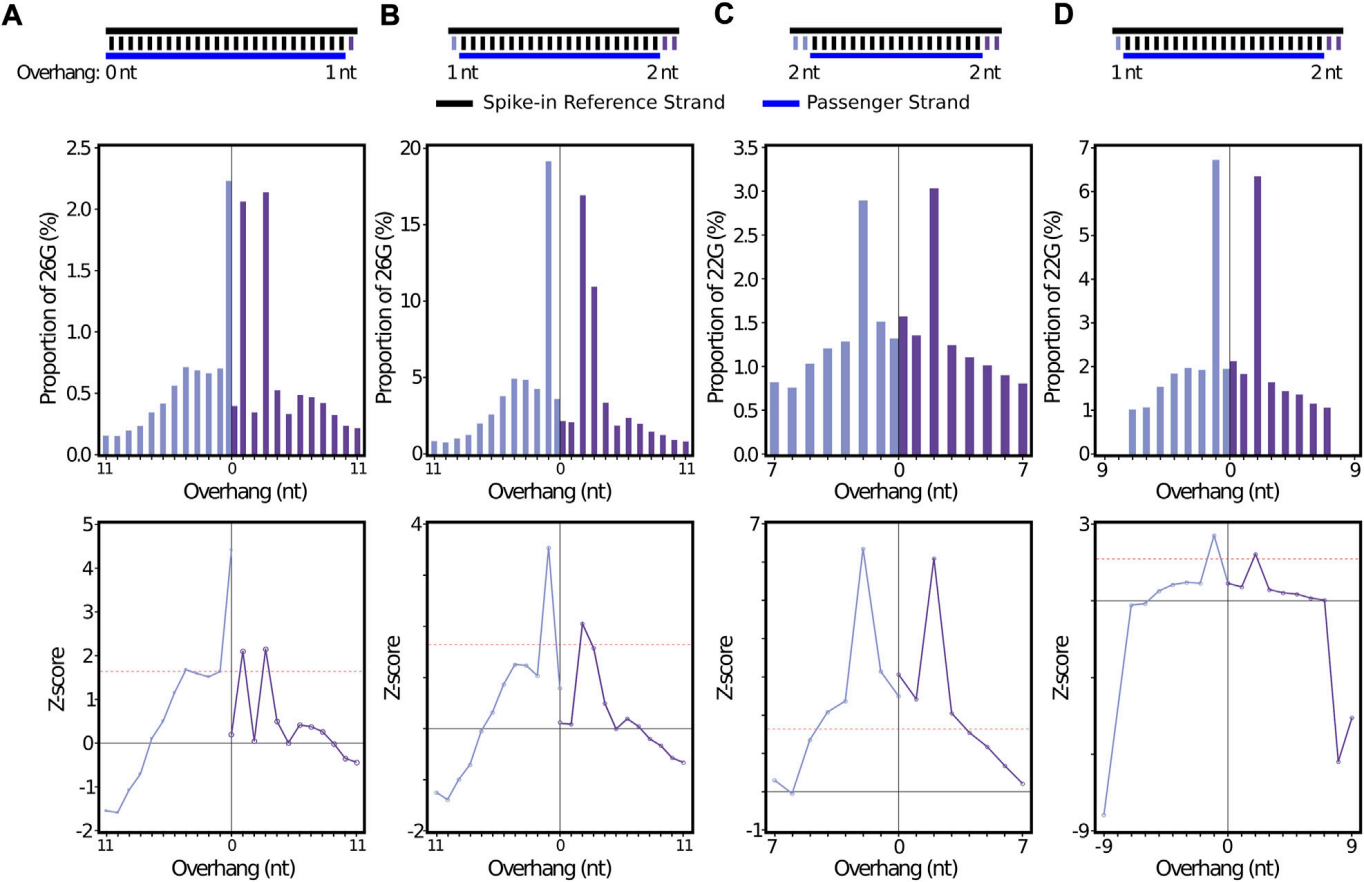
stepRNA can identify an *in silico* sRNA spike-in dataset in biological data. A total of 5000 simulated 24 nt sRNAs and their associated passenger sequences were added to the biological dataset used in Figure 3. The *in silico* spike-in duplexes, including the reference and passenger sequence length and overhang distances are illustrated above the bar graphs. A single reference sRNA read was randomly selected and spiked-in 5000x to the non-collapsed **(A)** 26G (n = 303,233) or **(C)** 22G (n = 302,979) data. For **(B and D)** non-identical 24-nt references were added to the collapsed **(B)** 26G (*n* = 33,651) or **(D)** 22G (n = 111,723) siRNA reads. Bar graphs represent the percentage of reads with an overhang length for the spiked-in 26G **(A and B)** or 22G **(C and D)** in a *C. elegans* sRNA biological data set (Fischer et al., 2011) (GEO: GSE32366). The 5′ overhangs are coloured light purple, 3′ overhangs are coloured dark purple. Z-scores are shown for all data sets. Red line indicates *p* = 0.05.

To provide additional *in silico* evidence that stepRNA is able to detect a Dicer cleavage signature, we created sRNA duplexes to add into the *C. elegans* datasets and then tested whether stepRNA could detect these ‘spike-ins’. For 26G and 22G reference spike-ins, stepRNA detected the spike-in sequences with the correct overhangs (Figures 7A,C). This was again supported by the Z-score output (Figures 7E,G, Supplementary Table S2G and H). The raw counts for the spike-in data identified the correct passenger lengths of 25 nt and 18 nt spike-in for 26G and 22G, respectively (Supplementary Table S2). This confirms that the correct overhangs are being uncovered by stepRNA, even within the noise of a biological dataset where there could be incorrect alignments.

Variable reference spike-ins show low off-target hits. To further validate the accuracy of stepRNA, we also used 5000 randomly generated 24 nt references that begin with either a 5′ C or A. These specifications were chosen as they are unlikely to have near matches to 26G or 22G reads. When the 24 nt references and passengers were added to the biological data, the expected peak of enriched overhangs was observed, with z-score confirmation (Figure 5B, D, F, G).

## 4 Discussion

With the recent increase in sRNA sequencing, particularly in non-model organisms, comes an increased demand for tools to analyse sRNA data, and a high-quality genome is often not available to the user. Characterisation of miRNAs has been well established through tools such as MirDeep2 (Friedländer et al., 2012), due to their high conservation between species and characteristic hairpin loop formation prior to maturation. However, classifying families of siRNA is more difficult, partly due to the lack of conservation of siRNAs between species. However, there are several common features specific to siRNAs that can be used to classify them including (i) sequence length, (ii) first 5′ nt, (iii) the Argonaute protein that the siRNA is loaded onto, and (iv) the mechanism by which the siRNAs are processed from precursor sequences, for example, by the Dicer enzyme. (i) and (ii) are easily identifiable using bioinformatic tools and are achievable through most of the currently available sRNA analysis packages e.g. Unitas, sRNApipe, sRNAnalyzer (Gebert et al., 2017; Wu et al., 2017; Pogorelcnik et al., 2018) can extract sRNAs based on their length or first nucleotide; (iii) is best achieved experimentally. Here, we have addressed (iv). We have developed a bioinformatic tool to identify Dicer cleavage signatures, which we have described and tested with simulated and biological datasets. Our pipeline expands upon currently available tools, providing more information about the sRNA duplexes that are cleaved by Dicer and allowing analysis to be carried out with raw sRNAseq data alone.

Dicer generates siRNAs by cleaving dsRNA to leave a shorter sRNA duplex with a signature overhanging sequence at the 3′ and 5′ ends of the sRNA guide sequence relative to the passenger sequence in the duplex. In *C. elegans* these are 2 nt and 4 nt at the 5′ and 3′ ends, respectively, and in *A. thaliana* both ends have a 2 nt overhang. The overhang features can be identified computationally, but most sRNA analysis tools are not designed to search for Dicer cleavage signatures. One exception is signature.py (Antoniewski, 2014), which searches a for a Dicer cleavage signature at the 3′ end of a dsRNA duplex, by first aligning sRNA reads to the genome then extracting the distance from the 3′ end of overlapping reads. However, this tool is limited by requiring a (high quality) genome assembly to align the sRNA reads, and it only outputs information about the overhang at the 3′ end i.e. no information about the 5′ end overhangs or passenger strand length. Another issue with using the genome coordinates alone, is that this method is unable to identify sRNAs which are derived from spliced precursor sequences such as exon-exon boundaries. Furthermore, there are problems that arise from a read that is able to multimap to more than one genomic loci and how the ‘correct’ mapping location is chosen. For example, signature.py recommends setting Bowtie (Antoniewski, 2014) to randomly select a mapping locus in if there is a multimapping sRNA read. This likely leads to sRNA duplexes being missed because two reads (a sRNA and the corresponding passenger strand) from the canonical duplex might align to different genomic locations and therefore the pairing is missed. siRNAs are commonly found to multimap to a genome (Johnson et al., 2016; Handzlik et al., 2020; Suleiman et al., 2022) and this is therefore an important consideration when identifying Dicer cleavage signatures. These short fallings were reflected in the results, and stepRNA outperformed signature.py on both simulated and biological datasets. Specifically, signature.py was unable to detect a clear Dicer cleavage signature in the datasets that we analysed, and the overall number of duplexes detected by signature.py was low for both simulated and real data sets. In our simulated dataset, 49% of sRNA reads multimapped to the genome between 2 and 6 times, and this limited the number of duplexes that could be identified by signature.py compared with stepRNA which can account for sRNAs that multimap to the genome (our simulated dataset did not have any sRNA reads that crossed exon-exon barriers, so this cannot contribute to the observed differences between stepRNA and signature.py).

Here, we have demonstrated that stepRNA is able to classify sRNAs, without the requirement of a genome assembly. This is achieved through aligning sRNAs directly to other sRNA sequences to identify the RNA sequences that putatively form duplexes, and then calculating the distance that one sequence overhangs or underhangs relative to the other sequence in the duplex. stepRNA plots these data to identify if there is a propensity for a particular overhang, indicating a Dicer cleavage signature. Furthermore, stepRNA calculates the frequency of passenger sequence lengths, and provides alignment files for downstream analyses. Because stepRNA requires only sRNA sequences it (i) is applicable to non-model organisms where limited or poor-quality genomic data is available *i.e.* only sRNAseq data is required, and (ii) accounts for sRNA duplexes that do not align directly to the genome sequence such as those originating from spliced precursor sequences such as span exon-exon junctions. stepRNA will simplify the discovery and characterisation of siRNA families from sRNAseq datasets and make results more reproducible through the incorporation of alignment and classification in a single tool.

Using simulated and real biological sRNA sequence data we have demonstrated that stepRNA is able to identify Dicer cleavage signatures and their passenger strand lengths for siRNAs of a known length and 5′ base e.g. the 26G siRNAs in *C. elegans*. stepRNA correctly found Dicer cleavage signatures of a 5′ 4 nt overhang and 3′ 3 nt overhang associated with 26G siRNA Dicer processing that have previously been experimentally confirmed (Blumenfeld and Jose, 2016). Also, we have shown that stepRNA can identify a candidate family of siRNAs that are cleaved by Dicer, and their passenger lengths, from using raw sRNA reads which include a range of sequence lengths and first 5′ base e.g. 24 nt siRNAs in *A. thaliana.* stepRNA was first run using all sRNA reads (18–28 nt) in a dataset and revealed a 24 nt siRNA was the most common sRNA to form a duplex. Dicer cleavage could then be confirmed by running stepRNA similar to how stepRNA was run on the *C. elegans* dataset using a 24 nt filtered reference input versus all sRNA reads. This showed that the expected 3′ 2 nt overhang was still identified. This agrees with experimental data that DCL3 generate sRNA duplexes with a 24 nt passenger that are the most abundant in *A. thaliana* (Meyer et al., 2015) and also data shown here from a *dcl2dcl3dcl4* mutant where a 22 nt passenger was instead the most common as expected due to DCL1 still being functional.

An unexpected finding that stepRNA uncovered was the large proportion of blunt ended sRNA duplexes in the *A. thaliana* when uncovering when either testing all sRNAs from a sRNA population against themselves for Dicer cleaved overhangs or running a more refined analysis using a 24 nt size selected sRNA of interest as reference sRNA as input. To our knowledge this has not been previously observed for 24 nt sRNA duplexes. We speculate this could be due to i) a novel sRNA duplex that could be a previously undetected sRNA class or ii) a secondary sRNA duplex product that is generated downstream of 24 nt Argonaute target RNA detection and breakdown that could be involved in the silencing of the target RNA by RdRM. It is unlikely that these blunt ends are the result of incorrect alignment of the short sequences in the stepRNA pipeline as we do not observe these blunt ends from the simulated or *C. elegans* datasets.

Because of the nature of the sRNA duplexes i.e. a passenger strand will undergo degradation at a faster rate than guide strands, it is possible that some passenger strands will not be sequenced. However, with stepRNA we have demonstrated that enough passenger strand sequences are present in sRNA data sets to pick up the Dicer cleavage signature. This is only therefore likely to be an issue for sRNA duplexes expressed at very low levels. stepRNA is designed to identify and predict canonical siRNA duplexes where the guide strand and the passenger strand are fully complementary (not including the overhang and underhang regions). Non-canonical siRNA duplexes i.e. those that do not have perfect complementarity between the guide and the passenger strand may also exist. While the stepRNA code used here uses a conservative approach and only identifies duplexes with perfect complementarity, the code can be easily modified at the discretion of the user to identify non-canonical duplexes or to account for sequencing errors (see Methods 2.5). Whilst increasing the number of mismatches permitted is expected to increase the number of duplexes detected in sequence data, this will also increase the potential for false positive identification of duplexes. Although not specifically addressed here, stepRNA could be adapted for application to any sequence data where the goal is to identify overlapping sequences in a double stranded sequences or a duplex. For example, in organisms where piRNA biogenesis involved the Ping-Pong cycle such as *Drosophila,* piRNAs overlapping in dual-strand clusters could be detected.

## 5 Conclusion

We have presented stepRNA, an easy to use tool that will facilitate the discovery of Dicer cleavage signatures in from sRNA read data without the requirement of a reference genome and will enhance the detection of siRNAs. stepRNA can be used to analyse siRNAs of known length and or first 5′ nt, or can be used with raw sRNA data to predict the sequences that are most likely to have a passenger sequence and Dicer cleavage signature. stepRNA is a tool that can be used to facilitate the characterisation of siRNA families using sRNA data alone.

## Data Availability

Publicly available datasets were analyzed in this study. This data can be found here: Gene Expression Omnibus (GEO): *C. elegans* (GSM801363); https://www.ncbi.nlm.nih.gov/geo/query/acc.cgi?acc=GSE32366 *A. thaliana* WT (GSM1845210) and *dcl2dcl3dcl4* mutant (GSM1845222); https://www.ncbi.nlm.nih.gov/geo/query/acc.cgi?acc=GSE71782.

## Supplementary material

Supplementary Figure S1 Underhang distances show no unexpected enrichment for any distances confirming 26G siRNAs are Dicer processed. stepRNA was run using collapsed **(A–D)** and non-collapsed **(E–H)** reference siRNAs reads against passenger sequence. Bar graphs, showing the percentage of references with an underhang, for the collapsed reads for **(A)** 26G (n = 28651) and b) 22G (n = 106723) siRNAs and line graphs showing the z-scores (**C and D**, respectively). For the non-collapsed, (e,g) 26G (n = 303232) and **(F and H)** 22G (n = 302979) show the percentage of references with an overhang and the corresponding z-score. 5’ overhangs are coloured light green. 3’ overhangs are coloured dark green. The red line represents P = 0.05, and ‘n’ equals the total number of reference reads input.

Supplementary Figure S2 Non-collapsed data can also identify the Dicer processing signature in 26G siRNAs. For the non-collapsed, **(A)** 26G (n = 303232) and **(B)** 22G (n = 302979) show the percentage of references with an overhang as bar graphs. The respective z-scores are shown for **(C)** 26G and **(D)** 22G as line graphs. 5’ overhangs are coloured light purple. 3’ overhangs are coloured dark purple. The red line is at p = 0.05 and n represents the total number of reference reads in File A.

Supplementary Figure S3
*A. thaliana* 5’ distances showed a weaker sRNA overhang signature. stepRNA was run using all sRNA reads **(A and B)** or 24nt sRNA reads **(C and D)** as reference input against all sRNA reads as query input. Expression was calculated using RPM and shown as a barplot against 5’ overhang length **(A and C)**. The percentage of sRNA reference reads with at least one query alignment is also shown as a barplot of 5’ overhang length against the percentage of reads **(B and D)** (n = 2908404 WT, n = 2733702 *dcl2dcl3dcl4* mutant).
