# Supplementary figures and images for: stepRNA: Identification of Dicer cleavage signatures and passenger strand lengths in small RNA sequences
Source: Front Bioinform. 2022 Nov 21;2:994871. doi: 10.3389/fbinf.2022.994871 (PMC9720893; doi:10.3389/fbinf.2022.994871)

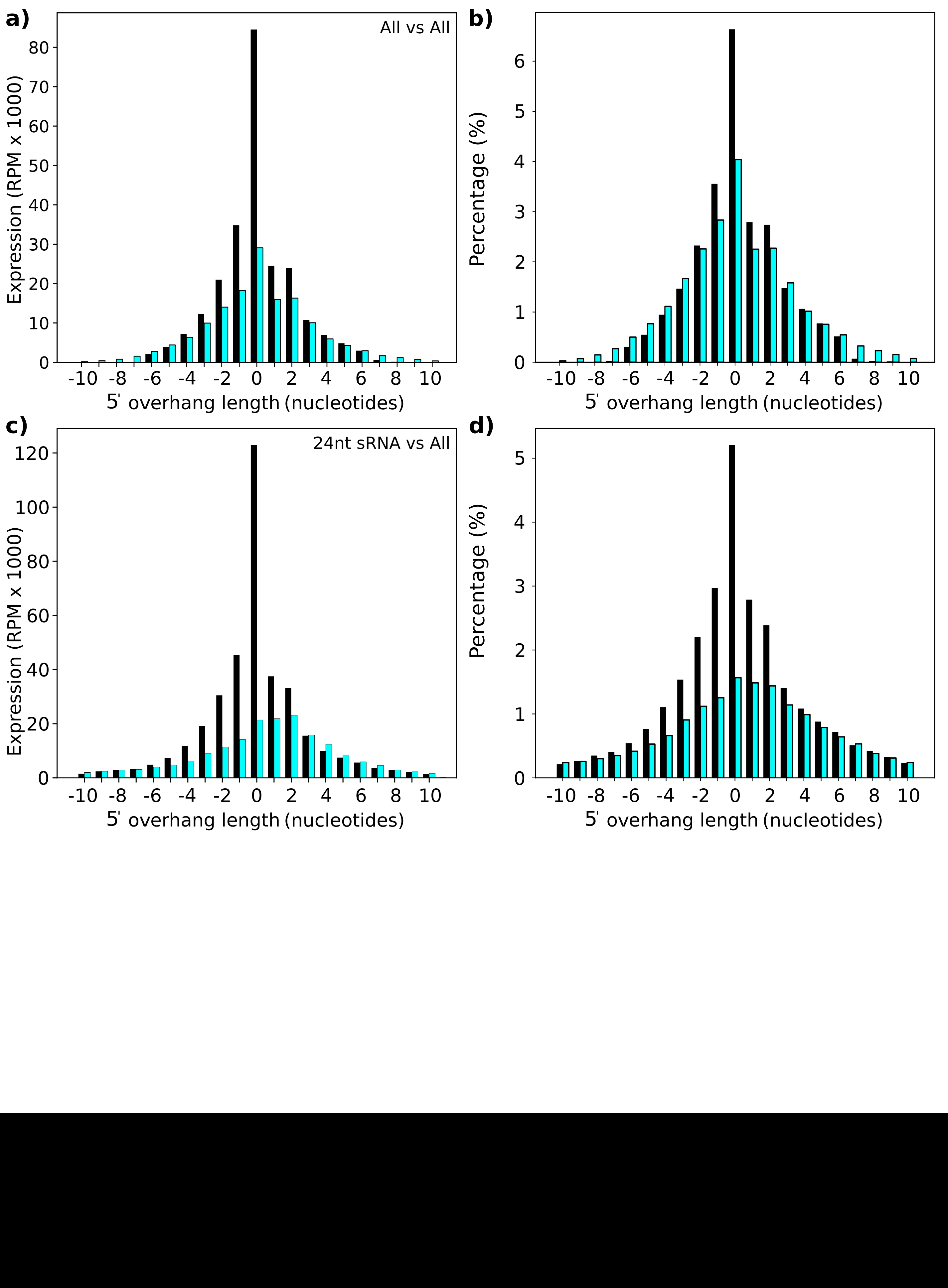

Supplement: Supplementary file 2 [file Image3.JPEG]

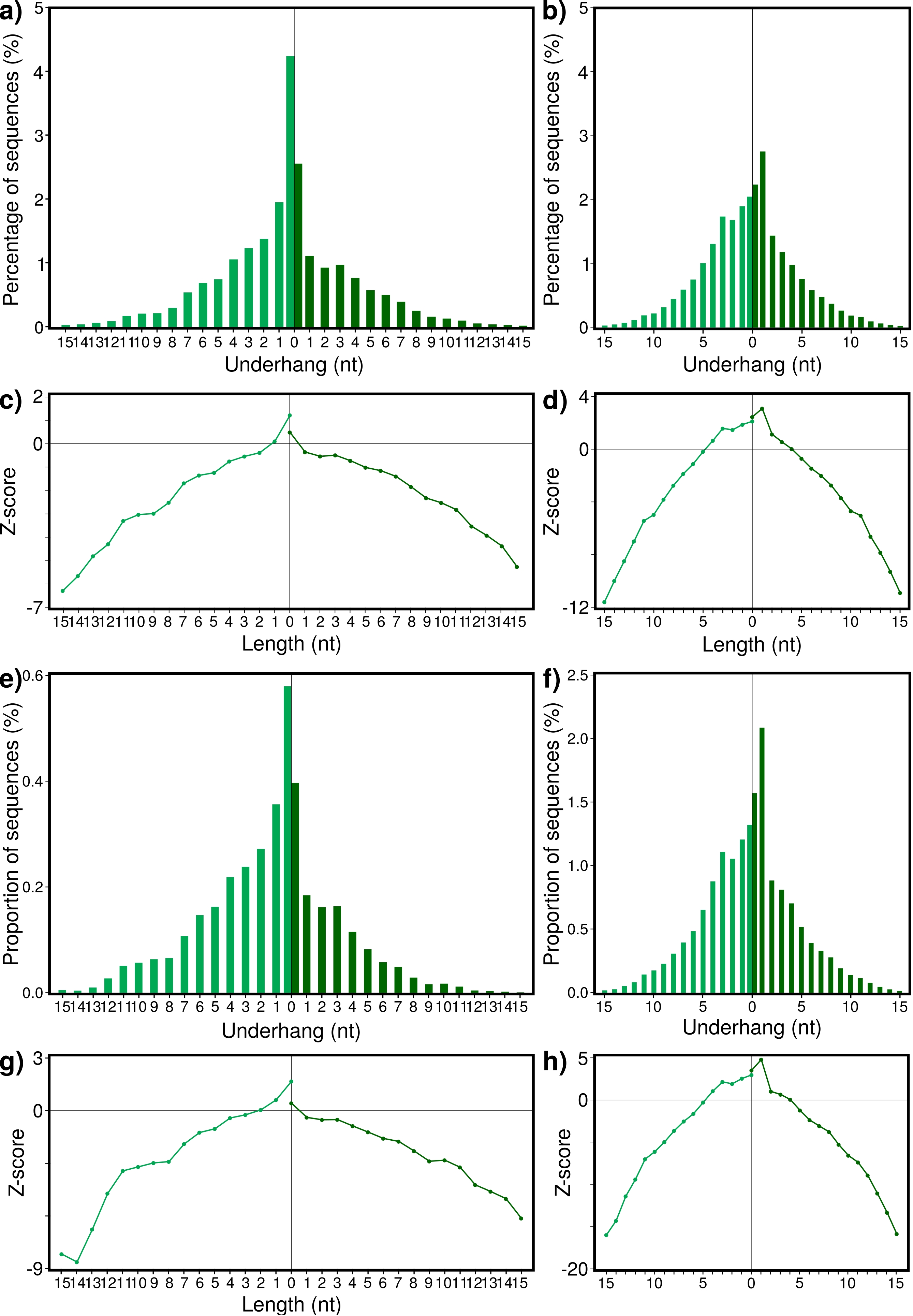

Supplement: Supplementary file 5 [file Image1.JPEG]

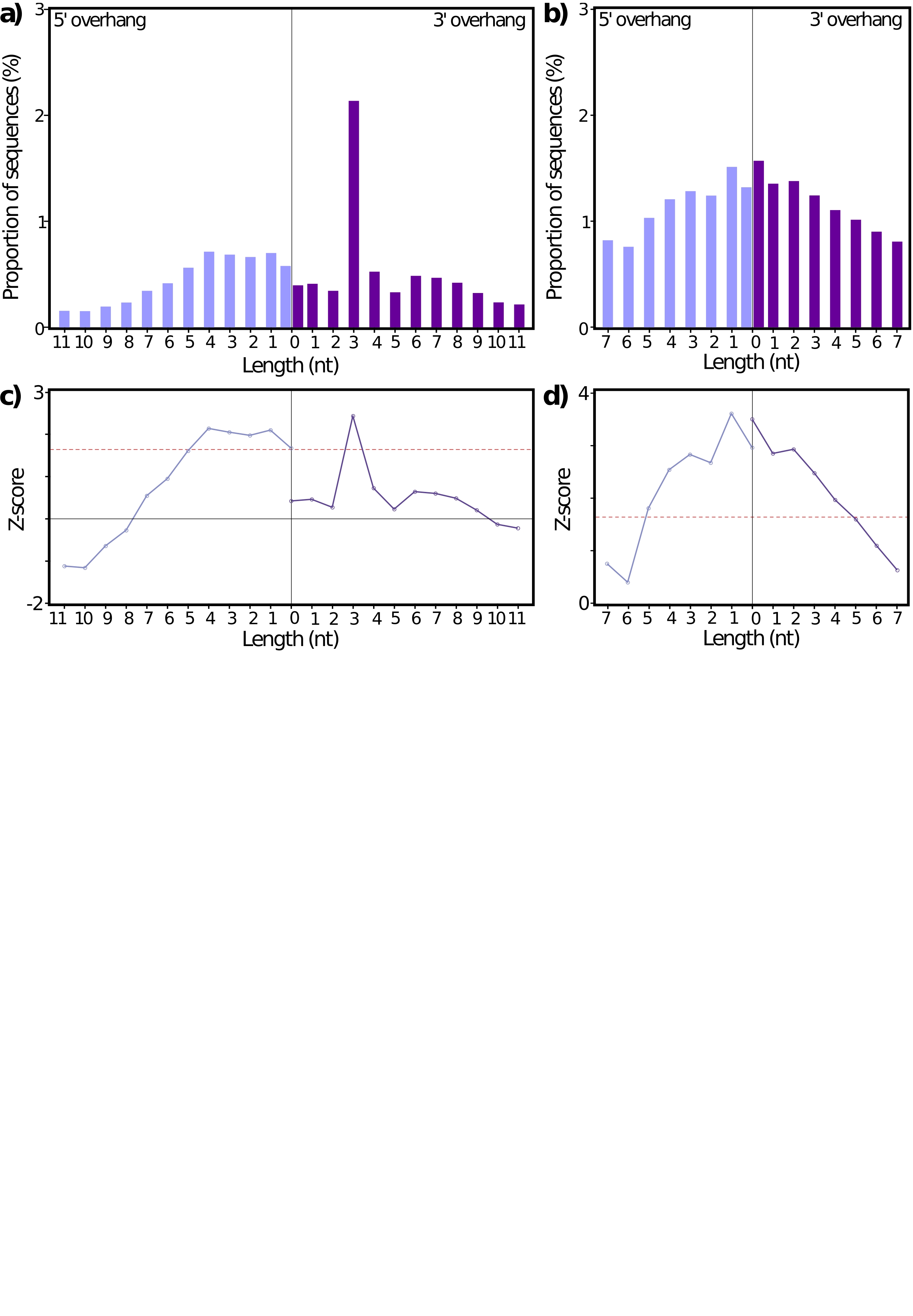

Supplement: Supplementary file 6 [file Image2.JPEG]
