# Supplementary material for: stepRNA: Identification of Dicer cleavage signatures and passenger strand lengths in small RNA sequences
Source: Front Bioinform. 2022 Nov 21;2:994871. doi: 10.3389/fbinf.2022.994871 (PMC9720893; doi:10.3389/fbinf.2022.994871)
Supplement: Supplementary file 4 [file Table1.DOCX]

| Multimapping occurrences for an sRNA from Dataset A | Number of sRNAs |
| --- | --- |
| 1 | 403 |
| 2 | 269 |
| 3 | 89 |
| 4 | 26 |
| 5 | 7 |
| 6 | 3 |

**Table A. summary of sequence loci occurrences in Dataset A**
