# Supplementary material for: stepRNA: Identification of Dicer cleavage signatures and passenger strand lengths in small RNA sequences
Source: Front Bioinform. 2022 Nov 21;2:994871. doi: 10.3389/fbinf.2022.994871 (PMC9720893; doi:10.3389/fbinf.2022.994871)
Supplement: Supplementary file 7 [file Table5.DOCX]

**Supplementary Table 5 | 26G sRNA duplex 3’ overhang counts for stepRNA against signature.py in *C. elegans***

| Overhang distance (nt) | stepRNA | signature.py |
| --- | --- | --- |
| -11 | 277 | 6 |
| -10 | 313 | 4 |
| -9 | 409 | 8 |
| -8 | 493 | 3 |
| -7 | 663 | 4 |
| -6 | 789 | 1 |
| -5 | 628 | 7 |
| -4 | 1132 | 5 |
| -3 | 3685 | 6 |
| -2 | 680 | 8 |
| -1 | 694 | 8 |
| 0 | 728 | 26 |
| 1 | 315 | 11 |
| 2 | 262 | 6 |
| 3 | 275 | 3 |
| 4 | 216 | 4 |

**Supplementary Table 6 | 22G sRNA duplex 3’ overhang counts for stepRNA against signature.py in *C. elegans***

| Overhang distance (nt) | stepRNA | signature.py |
| --- | --- | --- |
| -7 | 1189 | 44 |
| -6 | 1285 | 34 |
| -5 | 1528 | 43 |
| -4 | 1609 | 34 |
| -3 | 1836 | 52 |
| -2 | 2087 | 34 |
| -1 | 2045 | 56 |
| 0 | 2376 | 100 |
| 1 | 2927 | 45 |
| 2 | 1525 | 107 |
| 3 | 1251 | 39 |
| 4 | 1038 | 39 |
| 5 | 803 | 48 |
| 6 | 615 | 42 |
| 7 | 504 | 42 |
| 8 | 389 | 33 |

**Supplementary Table 7 | 24nt sRNA duplex 3’ overhang counts for stepRNA against signature.py in WT *A. thaliana***

| Overhang distance (nt) | stepRNA | signature.py |
| --- | --- | --- |
| -10 | 11742 | 52831 |
| -9 | 13109 | 52353 |
| -8 | 14596 | 54660 |
| -7 | 16970 | 55286 |
| -6 | 27978 | 56133 |
| -5 | 35263 | 57407 |
| -4 | 43305 | 57834 |
| -3 | 58106 | 56916 |
| -2 | 103799 | 59425 |
| -1 | 109209 | 62625 |
| 0 | 133990 | 59815 |
| 1 | 38614 | 62741 |
| 2 | 35396 | 59218 |
| 3 | 25713 | 60987 |
| 4 | 21319 | 61197 |

**Supplementary Table 8 | 24nt sRNA duplex 3’ overhang counts for stepRNA against signature.py in DCL234 mutant *A. thaliana***

| Overhang distance (nt) | stepRNA | signature.py |
| --- | --- | --- |
| -10 | 11457 | 7101 |
| -9 | 12271 | 6982 |
| -8 | 13435 | 7412 |
| -7 | 15123 | 7278 |
| -6 | 20198 | 7553 |
| -5 | 25228 | 7640 |
| -4 | 27344 | 7627 |
| -3 | 32754 | 7411 |
| -2 | 39591 | 7745 |
| -1 | 41937 | 7850 |
| 0 | 39479 | 8284 |
| 1 | 24577 | 8242 |
| 2 | 24545 | 7549 |
| 3 | 19387 | 8163 |
| 4 | 14893 | 8137 |
